# The Gut Microbiome in the IgE-Mediated Food-Allergic Patient—A Narrative Review

**DOI:** 10.3390/nu18040593

**Published:** 2026-02-11

**Authors:** Neel Singh, Erin Hosein, Yamini V. Virkud, Corinne Keet, Michael Kulis

**Affiliations:** 1 Department of Pediatrics, School of Medicine, University of North Carolina at Chapel Hill, Chapel Hill, NC 27599, USA; 2 Department of Nutrition, Gillings School of Global Public Health, University of North Carolina at Chapel Hill, Chapel Hill, NC 27599, USA; 3 Department of Anthropology, University of North Carolina at Chapel Hill, Chapel Hill, NC 27599, USA; 4 Department of Pediatrics, Massachusetts General Hospital, Boston, MA 02114, USA; 5 Harvard Medical School, Boston, MA 02115, USA

**Keywords:** food allergy, microbiome, microbiota, oral immunotherapy, food allergen immunotherapy, omalizumab, probiotic, fecal microbiota transplantation, immune supportive diet

## Abstract

Food allergies (FA) are a major public health concern in both children and adults. Immunoglobulin E (IgE)-mediated FA is characterized by allergic reactions driven by allergen-specific IgE and the subsequent degranulation of mast cells and basophils. Current FA management primarily involves avoidance of allergen-containing food, and more recently, therapies such as oral immunotherapy (OIT), sublingual immunotherapy (SLIT), and the anti-IgE biologic omalizumab. However, these interventions are not curative. The gut microbiome has been implicated in the development and regulation of oral tolerance to food antigens. This narrative review explores the role of probiotics, fecal microbiota transplantation (FMT), dietary interventions, and the interaction between the microbiome and OIT as potential strategies to manage established FA. We also explore barriers to their proliferation as part of regular clinical care. We conclude that future research should (1) address how the microbiome interacts with immunotherapies other than OIT, (2) explore the role of novel microbiome-based treatments like FMT as potential adjuvants to existing food allergy therapeutics, and (3) focus on developing standardized protocols and endpoints for microbiome-based therapeutics.

## 1. Introduction

Food allergies (FA) are a significant public health concern. In the U.S., food allergies are estimated to affect 8.0% of children and 10.8% of adults [1,2]. FA is commonly mediated by abnormal immunoglobulin E (IgE) production against food antigens and is characterized by a range of allergic symptoms, including hives, gastrointestinal upset, and potentially life-threatening anaphylaxis occurring within minutes to a few hours after exposure [3,4]. In contrast, non-IgE-mediated FA involves different immune mechanisms, such as T cell mediation, and symptom onset may be delayed [4,5]. IgE-mediated FA is established when the antigens of an allergen are detected by antigen-presenting cells (APCs). These APCs, which include dendritic cells, macrophages, and B cells, prompt naïve T cells to differentiate into T helper 2 (Th2) and T follicular helper (Tfh) cells. Th2 cells secrete the cytokines IL-4, IL-5, and IL-13 to promote inflammation and B cell activation [4]. Tfh cells also produce inflammatory cytokines and promote B cell production of IgE [4,6]. The next step in this pathway is the binding of allergen-specific IgE to mast cells and basophils via the FcεRI receptor, resulting in the sensitization of these granulocytes to the allergen. Re-exposure to these allergens results in the cross-linking of IgE on the surfaces of granulocytes, triggering rapid degranulation and the release of inflammatory mediators including histamine, leukotrienes, and prostaglandins. The release of these mediators results in the symptoms of IgE-mediated FA [4]. There is no cure for FA; however, traditional management for individuals with established FA relies on strict avoidance of allergen-containing foods, and more recently, allergen immunotherapies (oral or sublingual) and anti-IgE therapy (omalizumab) [4,7,8,9].

The microbiome is a “characteristic microbial community occupying a reasonable well-defined habitat which has distinct physio-chemical properties” [10]. This review focuses on the microbiome of the gut. A disrupted gut microbiome, or dysbiosis, has been linked to the development of FA. Delayed maturation of the gut microbiome during the first year of life has been linked to later onset of FA [11]. Given the link between the gut microbiome and FA, microbiome-based interventions are of interest to modify disease course after FA has already developed. In this narrative review, we examine how interventions that modulate the microbiome might help manage established FA, both as standalone treatments and as adjuvants to existing treatments. We discuss mechanisms of microbiome–immune interactions, various interventions (probiotics, fecal microbiota transplantation, diets, and food allergen immunotherapy), pre-clinical and clinical evidence from animal models and human trials (across infants, children, and adults), and barriers to the proliferation of these interventions as part of clinical care.

We began with a PubMed search using keywords relating to food allergy, the microbiome, probiotics, oral immunotherapy, and fecal microbiota transplantation. Articles from this PubMed search must have been published in 2010 or later. We supplemented this with a reference list screening of relevant literature and by including publications already recognized as influential. Because this is a narrative review, article inclusion was based on conceptual relevance rather than strict systematic criteria. Limitations of this review article include its narrative design and non-systematic article selection, which may introduce bias as quality and relevance were not assessed in a standardized manner.

## 2. Mechanisms of Gut Microbiome–Immune Interactions

**Figure 1:**
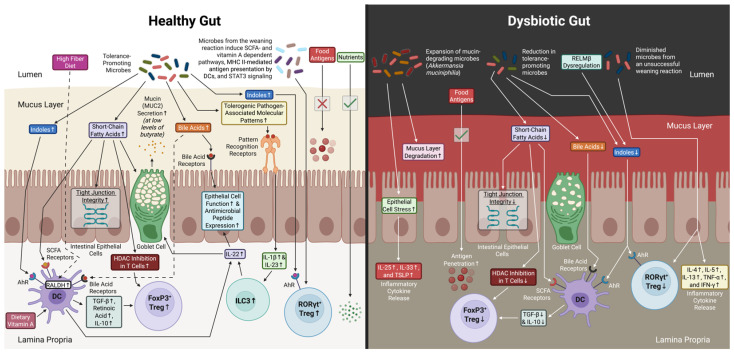
Sample of mechanisms relevant to food allergy that may underlie differences in a healthy gut (**left**) and dysbiotic gut (**right**). In a healthy gut, a high-fiber diet and healthy microbiome support the integrity of the gut mucus layer, short-chain fatty acid (SCFA) production, epithelial barrier function, and immune tolerance via FoxP3+ and RORγt+ regulatory T cells (Tregs) and group 3 innate lymphoid cells (ILC3). Dysbiosis disrupts the mucus layer, reduces SCFA production, and impairs barrier integrity, allowing for the translocation of antigens across the gut epithelium. This promotes the production of pro-inflammatory cytokines (IL-4, IL-5, IL-13, IL-25, IL-33, TSLP, IFN-γ, and TNF-α) while inhibiting the differentiation of Tregs. SCFA, Short-chain fatty acid. MHC II, Major histocompatibility complex class II. DC, Dendritic cell. HDAC, Histone deacetylase. RALDH, Retinal dehydrogenase. AhR, Aryl hydrocarbon receptor. Treg, Regulatory T cell. ILC3, Group 3 innate lymphoid cell. Original figure prepared in BioRender (Toronto, ON, Canada).

In healthy individuals, the intestinal epithelium allows for the absorption of nutrients while excluding potentially harmful macromolecules such as food antigens. The failure to maintain this selectivity may contribute to the development of FA [12]. The gut microbiome is important in preserving epithelial barrier integrity. In FA, it is hypothesized that dysbiosis can disrupt this barrier, leading to increased intestinal permeability and access of allergens to the immune system [13]. Figure 1 contains a graphical comparison of mechanisms relevant to FA that may underlie differences in a healthy gut and a dysbiotic gut.

In a healthy gut, tight junction complexes across intestinal epithelial cells (IECs) reduce the permeability of the gut epithelial layer. Goblet cells produce mucins that create a protective mucus layer [14]. IECs have pattern recognition receptors (PRRs) that detect pathogen-associated molecular patterns (PAMPs) including bacterial lipopolysaccharide, flagellin, and microbial cell wall components. Activation of these PRRs can trigger the release of cytokines, which act on immune cells beneath the gut epithelium [15]. Two cytokines, IL-1β and IL-23, have been shown in a human lamina propria mononuclear cell model to act on group 3 innate lymphoid cells (ILC3s), which are important for mucosal defense and repair. ILC3s produce IL-22 in response to IL-1β and IL-23, which has been noted by the authors to act on IECs to promote epithelial cell survival and proliferation, increase production of mucus, and increase the expression of antimicrobial peptide (AMP) genes [16]. AMPs are proteins that function as part of the innate immune response against harmful microbes [17].

The metabolism of dietary fiber by the gut microbiome produces the short-chain fatty acids (SCFAs) butyrate, acetate, and propionate, which have many immunomodulatory properties [18]. Butyrate, for example, has been shown in a Caco-2 cell monolayer model to promote the expression of tight junction proteins [19]. In human goblet cell-like cell line experiments, low levels of butyrate have been shown to stimulate expression of the mucin MUC2, which, as noted by the authors, may possibly elicit a protective effect on the epithelial barrier in vivo. However, high concentrations decrease MUC2 production and may lead to a diminished barrier function [20]. Mice lacking the SCFA receptors GPR43 and GPR109A had lower levels of tolerogenic CD103+ dendritic cells (DCs) and heightened FA symptoms. The authors believe this is suggestive of the importance of SCFA signaling in maintaining tolerance towards food antigens. Additionally, mice being fed a high-fiber diet showed a greater ability to induce FoxP3+ regulatory T cells (Tregs), which are important for maintaining immune tolerance, via upregulation of retinal dehydrogenase (RALDH) activity in CD103+ DCs. This effect was dependent on dietary vitamin A intake [21]. Butyrate has been shown in a splenic naïve CD4+ T cell model to promote the differentiation of naïve T cells into FoxP3+ Tregs. This is believed by the authors to occur through increased histone acetylation at the *Foxp3* locus, a process that butyrate can promote via histone deacetylase (HDAC) inhibition. The findings of the *in vitro* model were corroborated by a murine model [22]. In murine models, low-fiber conditions have been shown to increase levels of *Akkermansia muciniphila*. These changes were associated with a degraded mucus layer and a heightened allergic response. Additionally, fiber-deficient conditions were associated with increased transcription of the inflammatory cytokines IL-25, IL-33, and TSLP in certain regions of the gut [23].

A murine model found that weaning, or the transition to solid foods from milk during infancy, may induce a transition from an infancy-associated microbiome to an adult-associated microbiome composition characterized by an expansion of *Clostridia* and *Bacteroidia*. This “weaning reaction” also drives the development of RORγt+ Tregs, which are important for establishing immune tolerance [24]. Mechanistically, the authors note that this may function via SCFA- and vitamin A-dependent pathways similar to those described previously, presentation of bacterial antigens by major histocompatibility complex class II (MHC II) on DCs, and activation of signaling pathways involving the STAT3 transcription factor [24,25]. When the weaning reaction was disrupted by antibiotic usage, a greater presence of the inflammatory cytokines IL-4, IL-5, IL-13, TNF-α, and IFN-γ was observed. Taken together, the authors conclude that a disruption of the weaning reaction may increase susceptibility to later life immunopathologies [24].

The metabolism of dietary tryptophan by the gut microbiome into indole derivatives is another method by which the microbiome influences allergic sensitization. These indoles activate the aryl hydrocarbon receptor (AhR) on IECs and immune cells including DCs and innate lymphoid cells [26]. In a murine colitis model, activation of the AhR induced a tolerogenic DC phenotype characterized by the release of the anti-inflammatory cytokines IL-22, TGF-β, and IL-10. The latter two cytokines are characterized as promoting Treg differentiation [27]. In murine models, the goblet cell protein RELMβ was found to reduce levels of the indole-producers *Lactobacilli* and *Alistipes*, leading to a drop in indole metabolites noted by the authors to promote RORγt+ Tregs. The inhibition of RELMβ was shown to protect the mice from FA. These mice had levels of indole metabolites restored and a greater abundance of RORγt+ Tregs compared to mice with a higher expression of RELMβ. In children with FA, elevated serum RELMβ and reduced levels of indole derivatives in the stool were observed. The authors hypothesize that early-life RELMβ dysregulation may interfere with the weaning reaction and deplete the RORγt+ Treg population necessary for long-term oral tolerance [28]. Thus, a microbiome lacking the microbes needed to produce AhR-binding indole ligands may exacerbate FA.

Bile acids are another key class of immunomodulatory molecules. In addition to supporting lipid digestion and nutrient absorption, bile acids contribute to immune signaling. After synthesis in the liver, primary bile acids are secreted into the gut, where some are metabolized by the microbiome into secondary bile acids. Bile acids act on receptors that are expressed by IECs and immune cells such as macrophages [29]. Bile acid signaling has been linked with enhanced epithelial barrier integrity, increased AMP expression, and the promotion of tolerogenic DC phenotypes [30].

## 3. Probiotics

Probiotics are defined by the International Scientific Association for Probiotics and Prebiotics as “live microorganisms that, when administered in adequate amounts, confer a health benefit on the host” [31]. They are of interest as a possible treatment for allergic disease. Probiotics may promote a tolerogenic immune environment by competing with pathogenic microbes, enhancing epithelial barrier function, and by modulating immune cell function [32].

A 2024 systematic review and meta-analysis by Tan-Lim and Esteban-Ipac included five studies that examined the role of probiotics in mediating the acquisition of tolerance to food allergy in pediatric populations [33]. Four studies focused on children with cow’s milk allergy (CMA) and one on children with peanut allergy. For all four CMA studies, results were combined to generate a pooled effect estimate. This pooled effect estimate included participants with both IgE- and non-IgE-mediated CMA. No statistically significant benefit was shown in terms of acquisition of tolerance to cow’s milk. A summary of these studies can be found in Table 1. For peanut allergy, no pooled effect was estimated as only one study was included. This study was Loke et al.’s trial of probiotic peanut OIT, which is examined further in Section 6.3. Loke et al. did not show a significant effect of probiotic peanut OIT on the acquisition of tolerance to peanut [34].

When the four CMA studies were examined by probiotic strain, a significant benefit in terms of tolerance acquisition was shown for *Lactobacillus rhamnosus* GG (LGG). The relative risk of failure to acquire tolerance associated with the LGG strain was 0.41, indicating that the LGG strain may promote the acquisition of tolerance. The two studies that examined LGG in children with CMA were Berni Canani et al.’s 2012 [35] and 2017 [36] trials.

Berni Canani et al.’s 2012 randomized trial examined 55 infants ages 1–12 months with both confirmed IgE- and non-IgE-mediated CMA. Twenty-seven received up to 12 months of extensively hydrolyzed casein formula (EHCF) supplemented with LGG (n = 9 with IgE-mediated CMA, n = 18 with non-IgE-mediated CMA). Twenty-eight received up to 12 months of EHCF only (n = 12 with IgE-mediated CMA, n = 16 with non-IgE-mediated CMA). Those receiving EHCF supplemented with LGG had a higher probability of acquiring tolerance to cow’s milk at 6 and 12 months compared to infants receiving EHCF alone. Although a benefit was found for both IgE- and non-IgE-mediated CMA, this benefit was stronger for infants with non-IgE-mediated CMA [35].

Berni Canani et al.’s 2017 randomized controlled trial (RCT) examined 220 infants ages 1–12 months with confirmed IgE-mediated CMA. One hundred ten infants received up to 36 months of EHCF supplemented with LGG, while 110 received up to 36 months of EHCF alone. This trial found that infants with IgE-mediated CMA receiving combined EHCF and LGG had significantly higher rates of tolerance acquisition to cow’s milk at 12, 24, and 36 months compared to EHCF alone [36].

The two CMA studies that did not use the LGG strain were Hol et al. [37] and Cukrowska et al. [38]. The pooled effect estimate of both studies did not demonstrate a conclusive effect of probiotics on the acquisition of tolerance to cow’s milk [33]. Hol et al.’s RCT examined the use of 12 months of a mixture of *Lactobacillus casei* CRL431 and *Bifidobacterium lactis* Bb-12 in infants under 6 months of age with both confirmed IgE- and non-IgE-mediated CMA [37]. The probiotic mixture was administered with EHCF to 59 infants. The control group of 60 infants received EHCF only. Probiotic usage was not shown to have a statistically significant impact on the acquisition of tolerance to cow’s milk at 6 and 12 months [37].

Cukrowska et al.’s RCT examined 60 children less than 2 years old with both IgE and non-IgE-mediated CMA and atopic dermatitis. The authors administered a mixture of the strains *Lactobacillus casei* ŁOCK 0900, *Lactobacillus casei* ŁOCK 0908, and *Lactobacillus paracasei* ŁOCK 0919 daily to 29 participants for 3 months. The control group of 31 children received casein hydrolysate instead of the probiotic mixture. Participants were then followed for 21 more months, with 21 from the probiotic group and 19 from the control group completing this observation period. Throughout the course of the study, these infants were either on a dairy-free diet and/or breastfed. At the end of the observation period, no statistically significant difference in terms of acquisition of tolerance to cow’s milk was found between both groups. Please note that Cukrowska et al. was only available in Polish, and we used Google Translate (Google, Mountain View, CA, USA) to translate the article to English [38].

While the systematic review and meta-analysis by Tan-Lim and Esteban-Ipac demonstrated no statistically significant benefit of probiotic consumption for promoting the acquisition of tolerance to cow’s milk, the authors note that this finding is limited by a low certainty of evidence. The authors identified inconsistent findings between studies, a high risk of attrition bias in 3 of the studies, and an overall low precision due to wide confidence intervals including the null value of no effect [33]. Although the LGG strain was shown to be beneficial in terms of promoting acquisition of tolerance to cow’s milk, the generalizability of this finding is limited by there being only two LGG studies, both of which were performed by the same group and in similar populations (Italian children) [33,35,36]. However, the authors do note that Berni Canani et al.’s 2017 study is the only of the four included studies not appraised as being of high risk of bias [33,36]. The authors performed an additional subgroup analysis by length of treatment, finding that probiotic usage for at least 2 years is associated with an increase in the acquisition of tolerance to cow’s milk. However, only Berni Canani et al.’s 2017 study [36] and Cukrowska et al. [38] were included in this analysis. Cukrowska et al. did not find that their examined probiotic strains promoted the acquisition of tolerance to cow’s milk, and given its relatively smaller sample, it was underweighted in the pooled analysis compared to Berni Canani et al.’s 2017 study (14.7% weight versus 85.3% weight) [33,36,38]. Thus, the evidence included in this meta-analysis supporting probiotic usage for at least 2 years is weak given that it is driven by one single study examining one probiotic strain in one patient population [33,36].

Besides Loke et al.’s trial of probiotic peanut oral immunotherapy for peanut allergy [34], the studies examined were all focused on children with cow’s milk allergy [33]. This supports the need for further analysis of the effects of probiotics in managing allergies to foods other than cow’s milk. Additionally, further RCTs examining diverse probiotic strains and optimal dosing in different types of FA are needed to truly understand if and how probiotics can be used to manage FA.

**Table 1:** Summary of studies examining the acquisition of tolerance to cow’s milk.

Author (Year)	Study Design	Population/Sample Size	Intervention/Exposure	Comparison	Outcome of Interest	Main Findings
Hol et al. (2008) [37]	RCT	119 infants under 6 months of age with confirmed CMA (both IgE- and non-IgE-mediated)	Twelve months of EHCF supplemented with *Lactobacillus casei* CRL431 and *Bifidobacterium lactis* Bb-12 (n = 59)	EHCF with no probiotics for 12 months (n = 60)	Acquisition of tolerance to cow’s milk	Probiotic usage had no effect on the acquisition of tolerance to cow’s milk at 6 and 12 months.
Cukrowska et al. (2010) [38]	RCT	A total of 60 children under 2 years of age with confirmed CMA (both IgE- and non-IgE-mediated) and atopic dermatitis	Three months of oral *Lactobacillus casei* ŁOCK 0900, *Lactobacillus casei* ŁOCK 0908, and *Lactobacillus paracasei* ŁOCK 0919 each day (n = 29), with 21 completing the two-year follow-up	Casein hydrolysate with no probiotics for 3 months (n = 31), with 19 completing the two-year follow-up	Acquisition of tolerance to cow’s milk	No differences were found in terms of acquisition of tolerance to cow’s milk at 24 months.
Berni Canani et al. (2012) [35]	Randomized Trial	Fifty-five infants aged 1–12 months with confirmed CMA (both IgE- and non-IgE-mediated)	Up to 12 months of EHCF supplemented with LGG (n = 27, IgE-mediated allergy = 9 [33.33%])	Up to 12 months of EHCF only (n = 28, IgE-mediated allergy = 12 [42.9%])	Acquisition of tolerance to cow’s milk	Infants receiving EHCF supplemented with LGG were more likely to acquire tolerance to cow’s milk at 6 and 12 months compared to those receiving only EHCF. This trend was weaker in those with IgE-mediated CMA compared to those with non-IgE-mediated CMA.
Berni Canani et al. (2017) [36]	RCT	A total of 220 infants aged 1–12 months with confirmed IgE-mediated CMA	Up to 36 months of EHCF supplemented with LGG (n = 110)	Up to 36 months of EHCF only (n = 110)	Acquisition of tolerance to cow’s milk	Infants receiving EHCF supplemented with LGG showed higher rates of tolerance acquisition to cow’s milk at 12, 24, and 36 months.

CMA, Cow’s milk allergy. IgE, Immunoglobulin E. EHCF, Extensively hydrolyzed casein formula. LGG, *Lactobacillus rhamnosus* GG. RCT, Randomized controlled trial.

## 4. Fecal Microbiota Transplantation

Fecal microbiota transplantation (FMT) aims to restore a healthy microbiome through the administration of donor stool into the gut of the patient [39]. In fact, FMT has been shown to be more effective than probiotics in microbiome reconstitution in individuals recovering from antibiotics [40]. The most notable use case for FMT is in the treatment of recurrent *Clostridioides difficile* infection. Mechanistically, FMT reconstitution may improve health via the restoration of microbial cross-feeding networks, reintroduction of SCFA-producing bacteria, and introduction of non-living components directly from transplant material such as metabolites [39].

FMT is currently being explored as a treatment for FA. A murine model demonstrated that FMT from an infant with CMA led to heightened FA features in germ-free mice upon sensitization to cow’s milk compared to germ-free mice receiving stool from a healthy infant. This study suggests that the transfer of a CMA-associated microbiome increases susceptibility to allergic responses upon sensitization, while transfer of a healthy infant’s microbiome results in a relatively protective effect upon sensitization [41].

A phase I trial for oral encapsulated FMT in 15 peanut-allergic adults was first registered in 2016 and completed in 2021 (ClinicalTrials.gov NCT02960074) [42]. The goal of this trial was to assess the safety and tolerability of oral FMT in peanut-allergic adults. Ten of the participants were given oral encapsulated frozen FMT with no antibiotic pretreatment. Five of the 15 participants were given antibiotics prior to receiving FMT. The primary outcome is the presence of adverse events related to FMT over 12 months. Secondary outcomes include an evaluation of whether FMT with or without antibiotic pretreatment can increase the threshold needed to react upon challenge with peanut, changes in serum peanut-specific IgE and peanut specific skin prick test wheal size, and changes in fecal microbiome composition over 12 months [42]. Another phase II trial for fecal microbial transplantation therapy (MTT), a standardized form of FMT, was first registered in 2023 and is currently recruiting (ClinicalTrials.gov NCT05695261) [43]. This trial is limited to 37 children ages 12–17 years. Part A of this trial aims to assess the primary outcome of whether 28 days of MTT combined with Vancomycin and Neomycin antibiotic pretreatment can raise the reaction threshold to 300 mg of peanut protein (equivalent to one peanut kernel) during a double-blind, placebo-controlled food challenge (DBPCFC) after 28 days of treatment and again after 4 months following therapy initiation in 12 patients. A secondary outcome of Part A modifies the threshold of the primary outcome from 300 mg to 600 mg of peanut protein. A placebo arm exists, with 12 participants receiving placebo MTT and placebo antibiotics. Part B of this trial aims to enroll 13 participants who are already receiving maintenance peanut OIT. These participants will receive Vancomycin and Neomycin antibiotic pretreatment and 12 weeks of MTT while continuing maintenance OIT. However, there is no control group in Part B. Open label oral food challenges will be given at baseline, after 12 weeks of MTT and OIT, and 12 weeks after cessation of MTT and OIT. These will assess a secondary outcome of changes in peanut tolerance at the end of treatment and the primary outcome of durability of these changes after discontinuing treatment for 12 weeks. Both Parts A and B will assess the secondary outcomes of the safety and tolerability of the treatment alongside changes in immunological markers and fecal microbiome composition [43]. Results from both trials have not yet been released [42,43]. Summaries of these trials are in Table 2.

**Table 2:** Clinical trials investigating the role of FMT in food allergy (FA) management.

Trial	Study Design	Population/Sample Size	Intervention/Exposure	Comparison	Outcomes of Interest	Status
Evaluating the Safety and Efficacy of Oral Encapsulated Fecal Microbiota Transplant in Peanut Allergic Patients; Phase I (2016, ClinicalTrials.gov NCT02960074, Sponsor: Rima Rachid) [42]	Open-label, Non-randomized Trial	Fifteen adults ages 18–40 years with confirmed IgE-mediated peanut allergy	Oral encapsulated FMT over 1–2 days without antibiotic pretreatment (n = 10)	Oral encapsulated FMT over 1–2 days with antibiotic pretreatment (n = 5)	Primary: Safety and tolerability of FMT and antibiotic pretreatment Secondary: Changes in peanut reactivity threshold, peanut skin prick test, peanut-specific IgE, and fecal microbiome composition	Completed; results not yet released
Evaluating the Safety and Efficacy of Oral Encapsulated Microbiota Transplantation Therapy in Peanut Allergic Patients; Phase II (2023, ClinicalTrials.gov NCT05695261, Sponsor: Rima Rachid) [43]	Part A: RCT	Part A: Twenty-four participants ages 12–17 years with confirmed IgE-mediated peanut allergy	Part A: Vancomycin or Neomycin antibiotic pretreatment for 7 days followed by oral encapsulated MTT for 28 days (n = 12)	Part A: Placebo antibiotics for 7 days followed by placebo MTT for 28 days (n = 12)	Part A: Primary: Change in peanut reactivity threshold from less than 100 mg to 300 mg peanut protein Secondary: Change in peanut reactivity threshold from less than 100 mg to 600 mg peanut protein, safety and tolerability of MTT and antibiotic pretreatment, changes in peanut skin prick test, peanut-specific IgE, fecal microbiome composition, and other immune markers	Recruiting
	Part B: Open-label	Part B: Thirteen participants ages 12–17 years with confirmed IgE-mediated peanut allergy undergoing maintenance peanut OIT	Part B: Vancomycin or Neomycin antibiotic pretreatment for 7 days followed by 12 weeks of MTT while continuing existing maintenance peanut OIT (n = 13)	Part B: None	Part B: Primary: Durability of changes in peanut reactivity threshold 12 weeks after treatment discontinuation Secondary: Change in peanut reactivity threshold at the end of treatment, safety and tolerability of MTT and antibiotic pretreatment with peanut OIT, and changes in peanut skin prick test, peanut-specific IgE, fecal microbiome composition, and other immune markers	

FMT, Fecal microbiota transplantation. IgE, Immunoglobulin E. RCT, Randomized controlled trial. MTT, Fecal microbial transplantation therapy. OIT, Oral immunotherapy.

## 5. Dietary Modulation of the Microbiome

Vlieg-Boerstra et al. propose an “immune-supportive diet” meant to assist with the management of FA [44]. Notably, this diet has not yet been vetted through completed trials. Rather, it is a theoretical framework meant to guide the development of dietary strategies to manage FA.

The inclusion of minimally processed plant-based foods, which are meant to promote a healthy microbiome, is key to the immune-supportive diet [44]. Dietary diversity (DD) is emphasized as a strategy to enhance microbial diversity, increase SCFA production, and support immune tolerance. While DD has received growing attention for its potential role in FA prevention during early life, particularly during complementary feeding, there is limited research on its use in allergy management [45,46]. One study found that children ages 8–27 months on a cow’s milk exclusion diet were more likely to have a lower overall DD, suggesting that restricted diets that result from avoidance of one or many foods may reduce microbial and nutritional exposures [47].

The diet also recommends fermented foods such as yogurt, kefir, tempeh, and sauerkraut for their ability to promote a healthy gut microbiome [44,48]. These foods may support mucin production and tight junction expression, reinforcing gut integrity [48]. In fact, a study comparing the effects of a high-plant fiber diet to a high-fermented food diet on the microbiome found that the high-fermented food diet performed better at increasing microbial diversity and decreasing inflammatory markers [49]. However, fiber is still recognized as a critical component of the immune-supportive diet due to its role in supporting the gut epithelial barrier [44].

Animal-based products are included in moderate proportions, with a preference for items from organic, free-range, or grass-fed sources (e.g., eggs, lean meat, poultry, and fatty fish) [44]. These sources provide higher omega-3 polyunsaturated fatty acid (PUFA) content and more favorable omega-6:omega-3 ratios, which have been linked to an anti-inflammatory immune environment [44,50]. The immune-supportive diet advises against ultra-processed foods, which often contain more food additives and emulsifiers than less-processed foods. These foods are known to promote dysbiosis and increase intestinal permeability [44,51,52]. Ultra-processed foods contain high levels of advanced glycation end products (AGEs), which are linked to a pro-inflammatory immune environment [53]. Certain food preparation methods, like steaming and boiling, may reduce AGEs compared to other methods, like grilling and roasting [44,54].

The proposed immune-supportive diet seeks to support the microbiome through a wide variety of plant-based foods and fermented products, promote the production of anti-inflammatory lipid mediators by including omega-3-rich sources, and strengthen epithelial barrier integrity via increased fiber consumption. At the same time, it minimizes exposure to microbiome-disrupting foods. This approach offers a potential adjunct to traditional allergen elimination strategies and therapeutics in the management of FA [44]. An ongoing RCT registered in 2022 is investigating the effect of a diet (similar to the immune-supportive diet proposed by Vlieg-Boerstra et al. [44]) on gut permeability, microbiome composition, co-occurring allergic symptoms, and quality of life in children with peanut and/or nut allergy (ClinicalTrials.gov NCT05667610) [44,55]. Extensive studies will be required to determine if these immune-supportive diets have any effect on both allergic outcomes and other measures of health.

## 6. Food Allergen Immunotherapy

Food allergen immunotherapy involves administering the protein of the food that the individual is allergic to in gradually escalating doses. The goal is to desensitize the individual and build tolerance towards the allergenic food through daily ingestion of the allergenic food. It has shown the ability to offer protection from accidental exposure by raising the threshold of reactivity to allergens, including peanut, milk, and egg. After years of OIT, some patients can achieve sustained unresponsiveness (SU), meaning that they can discontinue daily OIT dosing while still being able to tolerate the allergenic food for weeks or months. Some patients will acquire tolerance to the allergenic food, but only with regular OIT dosing. This is termed desensitization [9]. OIT dosing carries a risk of adverse events, which include gastrointestinal symptoms or even anaphylaxis [56,57]. Other forms of food allergen immunotherapy include sublingual immunotherapy (SLIT) and epicutaneous immunotherapy (EPIT) [58,59]. However, these therapies are not well-studied in the context of the microbiome. We examine various clinical research at the intersection of OIT and the microbiome, all summarized in Table 3.

### 6.1. The Microbiome and OIT

In multiple studies, successful OIT has been associated with a shift in microbiome composition, with conflicting evidence supporting changes in microbial diversity. Microbial diversity is generally measured in two forms: α-diversity, which measures within-sample diversity using a variety of measures (e.g., richness, Shannon index, and evenness), and β-diversity, which measures between-sample diversity [60].

He et al. analyzed fecal samples from nine participants of an RCT on peanut OIT [61]. Seven participants, aged 22–49 years, received peanut OIT, while two received placebo oat flour. Microbiome changes were analyzed between baseline and 52 weeks of treatment, with a focus on the changes induced by OIT. Among OIT participants, α-diversity significantly increased between baseline and 52 weeks across multiple measures (richness, Shannon index, and Pielou’s evenness). There was no consistent trend in β-diversity from baseline to 52 weeks among OIT participants. However, β-diversity did differ significantly between individuals. Compared to baseline, seven bacterial species were present at a greater abundance at 52 weeks among OIT participants. These include five members of the class Clostridia (including the families Oscillospiraceae, Lachnospiraceae, and Ruminococcaceae), one bacterium from the Firmicutes phylum, and one species from the *Bacteroides* genus [61]. The authors noted that many of the species from the families in the Clostridia class listed previously have been associated with tolerogenic immune modulation and protection from FA in murine models [61,62]. The species *Clostridium* sp. chh4-2 was decreased between baseline and 52 weeks of OIT [61].

Blackman et al. analyzed stool and buccal microbiome profiles in 17 peanut-allergic children ages 4–15 years undergoing peanut OIT (albeit with no placebo group) [63]. These children took approximately 40 weeks to meet the 300 mg maintenance dose from the 2 mg starting dose. Samples were taken one month after starting maintenance, meaning that 44-weeks of OIT dosing separate the pre- and post-OIT timepoints. For stool samples, α- and β-diversity showed no differences between pre- and post-OIT timepoints. Buccal samples, however, showed significant increases in Shannon index and Pielou’s evenness between pre- and post-treatment. Changes in richness were not significant. The authors posit that these differences in microbiome changes between the stool and buccal samples may result from peanut being “first encountered immunologically by the oral mucosa.” The authors also examined stool SCFA levels, which we will discuss in Section 6.2 [63].

A study of 32 school-age children with CMA undergoing milk OIT performed by Shibata et al. examined changes in the fecal microbiome associated with achieving SU [64]. There was no placebo control. Four children dropped out before SU could be assessed. The seven children who developed SU following a two-week milk avoidance period had higher levels of bacteria from the Bifidobacteriaceae family, which includes *Bifidobacterium*, at baseline. While immunological markers, like IgE levels, improved in all children on therapy, *Bifidobacterium* showed an increase only in the SU group compared to those not achieving SU. The authors found that α-diversity changed initially amongst OIT participants but tended to shift towards baseline by the end of treatment. These findings suggest that at baseline, the gut microbiome composition in terms of *Bifidobacterium* abundance may serve as a biomarker of OIT efficacy alongside traditional immune markers like IgE. However, changes in microbiome composition induced by OIT may be transient or limited to specific genera [64].

Badolati et al. selected 17 peanut-allergic children undergoing a peanut OIT trial along with 17 placebo participants for a gut microbiome and plasma metabolome profiling of changes associated with OIT administration [65]. All children were between the ages of 1–3 years. Fecal samples from 15 children from each group were used for the microbiome analysis, and comparisons were made between baseline and one year of OIT or placebo. The α-diversity was generally higher in the OIT group. The β-diversity did not vary between the OIT and placebo groups. Like He et al. [61], the authors found an elevated abundance of members of the Clostridia class in the OIT group compared to the placebo group. The metabolomic insights from this study will be discussed in Section 6.2 [65].

A study by Özçam et al. analyzing 90 children ages 1–4 years enrolled in a peanut OIT trial identified differences in the gut microbiome between those who achieved SU compared to those who did not [66]. Sixty-seven children underwent peanut OIT for 134 weeks, then stopped treatment while avoiding peanut for 26 weeks to assess for SU. Twenty-three children received placebo oat flour for the same duration. Özçam et al. did not find significant differences in α-diversity or β-diversity in fecal microbiome composition over time between placebo and OIT participants, suggesting that peanut OIT did not significantly modify the microbiome. However, participants achieving SU had lower baseline α-diversity and a distinct β-diversity profile compared to those not achieving SU [66]. In the parent clinical trial, lower baseline concentrations of peanut-specific IgE predicted SU status [67]. Özçam et al. found that α-diversity and total IgE, peanut-specific IgE, and Ara h 2-specific IgE were positively correlated at baseline. The authors note that these associations suggest that increased pre-OIT α-diversity is associated with higher pre-OIT IgE levels and reduced likelihood of achieving SU. Next, the authors identified that higher pre-OIT levels of *Romboutsia ilealis* and *Romboutsia timonensis* were linked with SU. However, *Ruminococcaceae*, *Parabacteroides distasonis*, and *Oscillospirales* were linked with failure to achieve SU [66].

Bouabid et al. included 37 children ages 3 months to 14 years [68]. Thirty had single or multiple IgE-mediated FAs and were undergoing OIT to various food allergens, including nuts, legumes (including peanuts), egg, milk, fish, and shellfish. These participants underwent OIT with a dosing build-up phase for 3–12 months followed by an 18-month maintenance phase at a 400 mg dose. Seven healthy children without FA were included as controls. These controls received no intervention. The study design did not allow for the assessment of SU. The authors performed fecal microbiome profiling. Amongst children with FA, no changes in α-diversity were observed between pre- and post-OIT. At baseline, higher richness, Chao1, and Shannon indices were observed in children with FA compared to healthy controls. A small, but significant change in β-diversity was observed between pre- and post-OIT. Additionally, at baseline, β-diversity was different between children with FA and healthy controls. Following OIT, Bacteroidota and Verrucomicrobiota abundance in children with FA was observed to shift towards the abundance seen in healthy controls. The authors believe that this finding is suggestive of OIT “normalizing the abundance” of certain phyla towards that of children without FA [68].

Across the examined studies, the impact of OIT on microbiome composition remains inconclusive. However, the abundance of specific taxa at baseline may serve as biomarkers of OIT success. Shibata et al. found that higher levels of *Bifidobacterium* were associated with SU [64]. Özçam et al. found that higher pre-OIT levels of *Romboutsia ilealis* and *Romboutsia timonensis* were linked with SU, while *Ruminococcaceae*, *Parabacteroides distasonis*, and *Oscillospirales* were linked with failure to achieve SU [66]. Thus, while evidence is mixed as to whether OIT remodels the overall microbiome, baseline microbiome composition specific to certain taxa may influence OIT response.

### 6.2. Gut-Associated Metabolites and OIT

Metabolomic profiling represents another approach to examining the influence of gut-associated metabolites on OIT outcomes. Studies have examined the fecal and plasma metabolome in association with OIT. These studies have identified differences in metabolites that are modified by gut microbiota, including bile acids, short-chain fatty acids, acylcarnitines, and phosphotidylcholines in patients who receive OIT compared to placebo or in patients who develop SU compared to those who do not.

Alongside their analysis of the effects of milk OIT on the microbiome, Shibata et al. examined changes in the fecal metabolome [64]. Similar to the trends observed relating to the microbiome, metabolomic changes occurred only at the beginning of OIT and returned to baseline by the end of treatment. However, the authors identified a negative correlation between monosaccharides and milk- and casein-specific IgE. Higher levels of milk- and casein-specific IgE were associated with a lower probability of achieving SU. The authors note that many monosaccharides serve as energy sources for gut microbiota. Additionally, bacteria of the Lachnospiraceae family were also associated with lower milk- and casein-specific IgE [64]. *Fusicatenibacter saccharivorans* is a member of this family and was noted by the authors to be associated with reduced intestinal inflammation and the production of monosaccharides [64,69,70]. The authors believe that these findings suggest the importance of the mucus barrier in supporting the development of oral tolerance [64].

In addition to performing a gut microbiome analysis, Badolati et al. profiled the plasma metabolome of children receiving either peanut OIT or no OIT [65]. Out of the 17 in each group, 10 children were randomly selected for metabolomic profiling. The authors found that levels of acylcarnitines and fatty acids generally increased over time in the OIT group and decreased in the placebo group. Additionally, lysophosphatidylcholines, a type of lipid, were found to be elevated post-OIT alongside uridine, pseudouridine, and bilirubin. Trigonelline was lowered in children receiving OIT post-treatment. Notably, plasma SCFA levels did not significantly differ between the OIT and placebo groups, though the authors note that metabolomic profiling of the stool and with a larger sample could yield differing results [65].

Complementing their oral and fecal microbiome analysis, Blackman et al. analyzed the SCFA levels in the stool of children undergoing peanut OIT [63]. The authors found increases in median fecal SCFA levels, though not statistically significant, between baseline and post-OIT. There did not seem to be a consistent trend in SCFA trajectories among participants, with some participants experiencing large post-OIT increases in SCFAs and others experiencing decreases [63].

Virkud et al. performed a metabolomic analysis of 20 children aged 7–13 years undergoing a trial of peanut OIT, and was the first to study the plasma metabolome with regard to SU [71]. Participants received OIT for 56 weeks, then underwent a 4-week avoidance period to assess SU. Significantly higher levels of most bile acids were observed in children failing to achieve SU. However, lithocholate and glycolithocholate were significantly higher in those achieving SU at baseline. Lithocholate levels remained relatively constant over the course of treatment, while glycolithocholate levels among those achieving SU and those failing to achieve SU were comparable after treatment. The authors note that some lithocholate metabolites have been shown in murine models to block the differentiation of pro-inflammatory Th17 cells and promote Treg proliferation and function [71,72,73]. At baseline, Virkud et al. found that levels of the histidine metabolite urocanic acid were at similar levels in those achieving SU and those failing to achieve SU [71]. However, post-OIT levels of urocanic acid were significantly elevated in those achieving SU. The authors note that urocanic acid has been shown to promote tolerance through increased IL-10 expression, decreases in the levels of the pro-inflammatory cytokine IFN-γ, and via the promotion of Tregs [74,75]. Succinic acid, which inhibits the production of urocanic acid, was found to be elevated in children failing to achieve SU. Like bile acid metabolism, succinic acid and urocanic acid metabolism were noted by the authors as being impacted by the microbiome [71]. *Achromobacter liquidum*, for example, has been shown to convert L-histidine to urocanic acid [76]. Finally, though not placebo-controlled, the authors studied the changes in metabolite profiles pre- and post-OIT, finding decreases over time in omega-3 and omega-6 PUFAs, another metabolite class that is modified by gut microbiota [71].

Adding to their profiling of the fecal microbiome, Özçam et al. profiled the fecal metabolome of participants and found that five bile acids derived from the gut microbiota and present in the stool prior to treatment predicted OIT success, suggesting that a specific microbiome composition may align with better performance on OIT [66]. These bile acids were 3-dehydrocholate, chenodeoxycholate, catechol sulfate, 7-ketolitocholate, and 7-ketodeoxycholate. This work supports the findings of Virkud et al., indicating that the bile acid profile could serve as a potential biomarker of OIT success [66,71].

The authors also found that children on OIT who did not achieve SU showed fecal microbiomes with pathways enriched for gluconeogenesis and anaerobic metabolism [66]. This was associated with the decreased fecal amino acid concentrations found in children who failed to achieve SU, suggesting that these children possess microbiomes with an enhanced capacity for amino acid metabolism. This enhanced amino acid metabolism in those not achieving SU could also lead to increased degradation of peanut antigens, which could potentially deplete the antigen exposure to the immune system necessary to build tolerance. This suggests that a microbiome that promotes degradation of peanut antigens could contribute to poor OIT outcomes [66].

### 6.3. Probiotics as Adjuvants to OIT

Studies have examined the effect of supplementing peanut OIT with probiotics. This combined therapy is called probiotic peanut oral immunotherapy (PPOIT). An RCT of 62 peanut-allergic children aged 1–10 years undergoing either 18 months of a combination of the probiotic strain *Lactobacillus rhamnosus* CGMCC 1.3724 and peanut OIT or placebo probiotics and placebo OIT was performed by Tang et al. [77]. In the PPOIT group, 82.1% of participants achieved SU, which was significantly higher than the 3.6% achieving SU in the placebo group. Significant reductions in skin prick test wheal sizes and peanut-specific IgE levels were observed alongside significant increases in peanut-specific IgG4 compared to baseline. Importantly, this study did not compare PPOIT to an OIT-only group, making it difficult to judge the impact of probiotics on participant outcomes [77].

A follow-up RCT of 201 children aged 1–10 years was performed by Loke et al. and included three arms: 18 months of the probiotic strain *Lactobacillus rhamnosus* ATCC 53103 and peanut OIT (n = 79), placebo probiotic and peanut OIT (n = 83), and placebo probiotic and placebo OIT (n = 39) [34]. The rate of SU for the PPOIT group was 46%. This was not significantly different from the 51% rate in the OIT-only group. However, both active arms showed significantly higher rates of SU compared to the 5% rate in the placebo group. Although the trial did not demonstrate that the addition of probiotics increased the rate of SU, there is some evidence that it improved safety. Children aged 1–5 years in the PPOIT group had a reduced incidence of adverse events compared to those in the OIT-only group. For children aged 6–10 years, the evidence is mixed [34].

Together, these trials suggest that while probiotics may not be effective as adjuvants to OIT, they may confer an improved safety profile in younger children. However, further trials are necessary to confirm this effect. An OIT trial registered in 2019 for egg allergy using *Lactobacillus rhamnosus* CGMCC 1.3724 as an adjuvant is ongoing (Australian New Zealand Clinical Trials Registry Number ACTRN12619000480189) [78]. This RCT has no egg OIT-only arm, meaning that the impact of probiotics on participant outcomes likely cannot be disentangled from that of egg OIT [79].

**Table 3:** Clinical research examining the interaction between food allergen immunotherapy and the gut microbiome.

Author (Year)	Study Design	Population/Sample Size	Intervention/Exposure	Comparison	Outcome(s) of Interest	Main Findings
Tang et al. (2015) [77]	RCT	Sixty-two children ages 1–10 years with confirmed peanut allergy	Combined *Lactobacillus rhamnosus* CGMCC 1.3724 and peanut OIT for 18 months up to a 2000 mg maintenance dose (n = 31)	Placebo probiotic and placebo OIT (n = 31)	SU and desensitization after 2–5 weeks of discontinuing treatment; changes in peanut skin prick test, peanut-specific IgE and peanut-specific IgG4	Amongst those receiving the combined probiotic and peanut OIT treatment, 82.1% achieved SU and 89.7% achieved desensitization. Amongst those receiving placebo probiotic and placebo OIT, 3.6% achieved SU and 7.1% achieved desensitization. The differences between both groups for SU and desensitization were significant. Those receiving combined probiotic and peanut OIT treatment had decreased peanut skin prick test wheal sizes and peanut-specific IgE, while peanut-specific IgG4 increased.
He et al. (2021) [61]	Retrospective analysis of an RCT	Nine patients with confirmed peanut allergy participating in a prior peanut OIT trial	Fifty-two weeks of peanut OIT up to a 4 g peanut protein maintenance dose (n = 7, ages 22–49 years)	Fifty-two weeks of placebo oat flour (n = 2, no age reported)	Fecal microbiome composition changes	α-diversity increased among OIT participants while no clear trend existed for β-diversity. Amongst OIT participants, species from five members of the Clostridia class, one bacterium from the Firmicutes phylum, and one species from the *Bacteroides* genus were elevated at the end of OIT dosing compared to baseline. *Clostridium* sp chh4-2 was decreased after treatment.
Loke et al. (2022) [34]	RCT	Two hundred one children ages 1–10 years with confirmed peanut allergy	Combined *Lactobacillus rhamnosus* ATCC 53103 and peanut OIT for 18 months up to a 2000 mg maintenance dose (n = 79)	Placebo probiotic and peanut OIT (n = 83) and both placebo probiotic and placebo OIT (n = 39)	SU after 8 weeks of discontinuing treatment	Amongst those receiving the combined probiotic and peanut OIT treatment, 46% achieved SU. Amongst those receiving placebo probiotic and peanut OIT, 51% achieved SU. Amongst those receiving placebo probiotic and placebo OIT, 5% achieved SU. SU rates for the combined probiotic and peanut OIT group and the placebo probiotic and peanut OIT group were not significantly different, though both were significantly higher than that of the placebo probiotic and placebo OIT group.
Blackman et al. (2022) [63]	Single-arm observational study	Seventeen children aged 4–15 years with confirmed peanut allergy undergoing peanut OIT	44 weeks of peanut OIT up to a 300 mg maintenance dose	Pre- versus post-OIT	Oral and fecal microbiome composition and fecal SCFA changes	Fecal samples showed no changes in α- or β-diversity. Buccal samples showed significant increases in the Shannon index and Pielou’s evenness between treatments, but not in richness. Median fecal SCFA levels did not significantly change between baseline and post-OIT.
Shibata et al. (2024) [64]	Ancillary study of a randomized trial	Thirty-two children ages 5–15 years with confirmed CMA participating in a cow’s milk OIT trial	Cow’s milk OIT for 13 months up to a 200 mL dose and a 2-week avoidance period	Participants achieving SU (n = 7) versus those not achieving SU (n = 21); 4 participants dropped out before SU could be assessed	Acquisition of tolerance to cow’s milk, immunological changes, and fecal microbiome and fecal metabolome changes	Only 22% of the original 32 participants achieved SU. Higher levels of bacteria from the Bifidobacterium family were associated with a higher likelihood of achieving SU after OIT. Microbiome changes were observed at the beginning of OIT, but returned to baseline by the end of treatment.
Virkud et al. (2024) [71]	Single-arm observational study	Twenty children aged 7–13 years with confirmed peanut allergy undergoing a peanut OIT trial	56 weeks of peanut OIT up to a 4 g maintenance dose and one month avoidance period	Participants achieving SU (n = 9) versus those not achieving SU (n = 11)	Plasma metabolome changes associated with OIT outcomes including SU	Children achieving SU had significantly higher levels of lithocholate and glycolithocholate at baseline compared to those not achieving SU. Urocanic acid was similar at baseline between both outcome groups, but those achieving SU had elevated urocanic acid post-OIT. Succinic acid inhibits the production of urocanic acid and was elevated in individuals failing to achieve SU.
Badolati et al. (2025) [65]	Ancillary study of an RCT	Thirty-four confirmed peanut-allergic children aged 1–3 years undergoing a peanut OIT trial	Peanut OIT for one year up to a 285 mg peanut protein maintenance dose (n = 17)	No OIT group (n = 17)	Fecal microbiome composition and plasma metabolome changes	α-diversity was generally higher in the OIT group, while β-diversity was similar between OIT and placebo groups. Members of the Clostridia class were elevated in the OIT group. Acylcarnitine, fatty acids, lysophosphatidylcholines, uridine/pseudouridine, and bilirubin were elevated post-OIT, while trigonelline was lowered. Plasma SCFAs did not differ between groups.
Özçam et al. (2025) [66]	Secondary analysis of an RCT	Ninety children ages 1–4 years with confirmed peanut allergy undergoing a peanut OIT trial	Peanut OIT for 134 weeks up to a 2000 mg maintenance dose and a 26-week avoidance period (n = 67)	Placebo oat flour for 134 weeks (n = 23)	Fecal microbiome composition changes, plasma metabolome changes, and their association with OIT response	Peanut OIT did not alter microbiome composition; however, those achieving SU had lower baseline α-diversity and a differential β-diversity profile compared to those not achieving SU. Baseline levels of *Romboutsia ilealis* and *Romboutsia timonensis* were linked with SU, while *Ruminococcaceae*, *Parabacteroides distasonis*, and *Oscillospirales* were linked with failure to develop SU. The bile acids 3-dehydrocholate, chenodeoxycholate, catechol sulfate, 7-ketolitocholate, and 7-ketodeoxycholate predicted OIT success. Children who failed to achieve SU had microbiome profiles characterized by elevated amino acid metabolism.
Bouabid et al. (2025) [68]	Prospective observational study	Thirty-seven children ages 3 months to 14 years	Children with confirmed IgE-mediated FA (including multiple FAs) undergoing OIT targeting various food allergens (nuts, legumes [including peanuts], egg, milk, fish, and shellfish) with a dosing build-up phase of 3–12 months followed by an 18-month maintenance phase at a 400 mg dose (n = 30)	Healthy controls with no FA (n = 7) receiving no treatment	Fecal microbiome composition changes	No changes in α-diversity were observed between pre- and post-OIT. However, some differences in α-diversity were observed between children with FA and healthy controls, with children with FA having higher richness, Chao1, and Shannon indices. β-diversity was significantly different between pre- and post-OIT; however, this change was small. At baseline, β-diversity was different between children with FA and healthy controls. Post-OIT, Bacteroidota and Verrucomicrobiota abundance amongst children with FA were shown to return towards the abundance observed in healthy controls.

RCT, Randomized controlled trial. OIT, Oral immunotherapy. SU, Sustained unresponsiveness. CMA, Cow’s milk allergy. SCFA, Short-chain fatty acids. IgE, Immunoglobulin E. IgG4, Immunoglobulin G4. FA, Food allergy.

## 7. Barriers to Proliferation

### 7.1. Clinical and Logistical Barriers

Patient adherence is a major barrier to implementing microbiome-based interventions. Typically, probiotics and OIT must be consumed daily to maintain effectiveness [9,35,36,37,38]. This contrasts with biologics such as omalizumab, which typically require less frequent dosing [80]. Daily dosing of these treatments may pose a barrier to patients who struggle with medication adherence.

Dietary interventions rely on strong patient compliance and acceptance of the diet’s composition, which may not always be tolerable to the patient. The proposed immune-supportive diet promotes consumption of minimally-processed whole foods [44]. This contrasts with the processed diets rising in prevalence worldwide [81]. Children who are picky eaters may have trouble tolerating newly introduced immune-supportive foods. FA, which already restricts many foods from the diet, may further lower compliance [47].

FMT introduces its own logistical barriers. The availability of suitable donor stool may limit access to future FMT for FA. One 2022 study found that only 10% of potential stool donors passed screening and were eligible for use in FMT. Screening is costly, with over €64,000 spent on biochemical testing to detect only 38 suitable donors out of 393 potential donors. While this study was focused on four separate trials for different microbiome-linked diseases, similar challenges may exist for food allergy [82]. Until the cost of FMT decreases, the treatment may be out of reach for many patients. Additionally, FMT for some conditions, such as ulcerative colitis, may benefit from the matching of a healthy donor to the recipient based on microbial diversity, presence of beneficial microbes and pathways, and a lack of harmful microbes and pathways [83]. A similar matching process could be beneficial for the quality and safety of FMT for FA; however, it would likely lower the number of potential stool donors and decrease access to the treatment.

### 7.2. Safety Considerations

Probiotics and FMT have favorable safety profiles, however some caution is warranted. A systematic review of case reports and case series highlights a risk of complications stemming from probiotic usage such as sepsis in older adults, individuals with *Clostridioides difficile* colitis, antibiotic usage, and *Saccharomyces* infections. The evidence is limited to 93 participants. However, clinicians should still exercise caution if administering probiotics to individuals from these groups [84]. In 2014, an infant died from gastrointestinal mucormycosis. A U.S. Centers for Disease Control and Prevention investigation linked the death to the consumption of probiotics contaminated with the mold *Rhizopus* [85]. A report from 2019 highlighted the transmission of drug-resistant *E. coli* in two separate clinical trials in two elderly patients undergoing FMT. Both patients received stool from the same donor. This was fatal for one of the patients [86]. Care should be taken to ensure the quality of administered probiotics and FMT. Indeed, this is especially important for immunocompromised groups, who should not be excluded from the potential benefits of microbiome-based therapeutics. Physicians should also ensure that patients are aware of the potential safety risks of consuming over-the-counter probiotics in search of FA relief.

OIT has well-documented safety risks. Common adverse events include gastrointestinal symptoms (such as abdominal pain, vomiting, and nausea) and oropharyngeal itching [56,57]. Importantly, OIT is associated with a nontrivial risk of systemic reactions, such as anaphylaxis, with an estimated 0.7 reactions needing epinephrine per 1000 OIT doses during the dosage escalation phase [87]. Additionally, some patients develop eosinophilic esophagitis, necessitating discontinuance of OIT [56,57]. Other forms of food allergen immunotherapy (SLIT and EPIT) may be associated with more favorable safety profiles [58,59].

## 8. Conclusions

Recent evidence highlights the potential of targeting the microbiome for the treatment of FA. The regulation of the immune system is mechanistically linked to the composition and function of the gut microbiome. Probiotics, specifically the LGG strain, have shown potential in the treatment of CMA. However, evidence in support of other strains or for FA other than CMA is lacking [33]. Data from FMT trials are still pending, leaving a gap in evidence [42,43]. The immune-supportive diet may hold potential in treating FA, but like FMT, trial data is not yet available [44,55]. Of these interventions, OIT is the best established, being one of the only U.S. Food & Drug Administration-approved treatments for FA [88]. Evidence supports that OIT is linked to the microbiome, yet the exact mechanisms of this link are not fully established. Generally, the studies we reviewed in Section 6 support that microbiome composition at baseline may influence OIT outcomes. However, evidence is mixed on whether OIT influences the microbiome throughout the course of treatment.

To date, microbiome research in food allergen immunotherapy has largely centered on OIT, with limited attention given to other modalities such as SLIT and EPIT. Future studies should aim to elucidate how SLIT and EPIT influence the microbiome. Additionally, research should examine how microbiome composition may affect SLIT and EPIT outcomes, allowing for the creation of microbiome-based biomarkers of treatment response. These microbiome-based biomarkers could complement traditional immunological markers such as IgE [89,90].

The integration of OIT with FMT represents a compelling avenue for future research. The synergy of both therapeutics could potentially yield results beyond what either could achieve alone. While a current trial is examining this intersection, the study design is limited as there is a small sample size and no placebo control [43]. If preliminary outcomes from this trial show promise, larger RCTs would be especially informative and impactful. Non-immunotherapy approaches, such as omalizumab, may also benefit from combination with microbiome-based therapeutics. Importantly, current treatments like OIT and omalizumab primarily serve to reduce the risk of severe reactions from accidental exposures and have not demonstrated curative potential. It is conceivable that an early-life approach, which integrates immunotherapy, microbiome modulation, and biologics, could promote the remission of FA. Although this hypothesis remains speculative, it supports the need for further clinical trials to evaluate the quality and safety of these combined strategies.

These trials should also aim to inform development of standardized FMT protocols for FA. Standardized protocols are likely necessary to scale the reach of FMT in FA beyond clinical trials as regulators are less likely to approve a treatment that does not have standardized administration protocols. The two current FMT trials incorporate some measurement of changes in microbiome composition and traditional immunological markers like allergen-specific serum IgE [42,43]. However, the creation of standardized microbiome endpoints can allow for better comparisons between trials. For example, increases in α- or β-diversity and increases in the abundance of SCFA-producing taxa could serve as hypothetical microbiome endpoints. Just as nearly all FA treatment trials include changes in allergen-specific IgE as an endpoint, a microbiome-based endpoint would standardize the evaluation of microbiome-based FA treatments. Research should determine what effective microbiome endpoints look like in the context of FA. Ideally, microbiome-based endpoints would be shared between all microbiome-targeting FA treatments, allowing for standardized comparisons of microbiome impact between modalities.

## Data Availability

No new data were created in this work.

## Abbreviations

The following abbreviations are used in this manuscript:

AGE	Advanced glycation end products
AhR	Aryl hydrocarbon receptor
AMP	Antimicrobial peptide
APC	Antigen presenting cell
CMA	Cow’s milk allergy
DC	Dendritic cell
DD	Dietary diversity
EHCF	Extensively hydrolyzed casein formula
EPIT	Epicutaneous immunotherapy
FA	Food allergy
FMT	Fecal microbiota transplantation
HDAC	Histone deacetylase
IEC	Intestinal epithelial cell
IgE	Immunoglobulin E
IgG4	Immunoglobulin G4
ILC3	Group 3 innate lymphoid cell
LGG	*Lactobacillus rhamnosus* GG
MHC II	Major histocompatibility complex class II
MTT	Fecal microbial transplantation therapy
OIT	Oral immunotherapy
PAMP	Pathogen-associated molecular pattern
PPOIT	Probiotic peanut oral immunotherapy
PRR	Pattern recognition receptor
PUFA	Polyunsaturated fatty acid
RALDH	Retinal dehydrogenase
RCT	Randomized controlled trial
SCFA	Short-chain fatty acid
SLIT	Sublingual immunotherapy
SU	Sustained unresponsiveness
Tfh	T follicular helper
Th2	T helper 2
Treg	Regulatory T cell
